# Tapping the Bioactivity Potential of Residual Stream from Its Pretreatments May Be a Green Strategy for Low-Cost Bioconversion of Rice Straw

**DOI:** 10.1007/s12010-018-2751-1

**Published:** 2018-04-16

**Authors:** Xingxuan Chen, Xiahui Wang, Yiyun Xue, Tian-Ao Zhang, Jiajun Hu, Yiu Fai Tsang, Min-Tian Gao

**Affiliations:** 1 Shanghai Key Laboratory of Bio-Energy Crops, School of Life Sciences, Shanghai University, 99 Shangda Road, Shanghai, 200444 China; 2 Department of Architecture, Shanghai Academy of Fine Arts, Shanghai University, 99 Shangda Road, Shanghai, 200444 China; 3 Department of Science and Environmental Studies, The Education University of Hong Kong, Ting Kok, Hong Kong, China

**Keywords:** Rice straw, Antioxidant activity, Phenolic compounds, Pretreatment, Biorefinery

## Abstract

In this study, it was found that the residual stream from pretreatments of rice straw exhibited high antioxidant activity. Assays based on the Folin–Ciocalteu colorimetric method confirmed that the residual stream contained large amounts of phenolic compounds. Three antioxidant assays were employed to evaluate the bioactivity of the residual stream. Strong linear correlations existed among the release of phenolic compounds, saccharification efficiency, and antioxidant activity. The alkaline pretreatment provided a much greater release of phenolic compounds, especially phenolic acids, compared to the acid pretreatment, and consequently, it had stronger linear correlations than the acid pretreatment. Antibacterial experiments demonstrated the ability of the phenolic compounds in the residual stream to inhibit the growth of microorganisms, indicating the potential of these compounds as antimicrobial agents. To discuss the possibility of the co-production of antimicrobial agents and biofuels/biochemicals, both acid and alkaline pretreatments were optimized using response surface methodology. Under the optimal conditions, 285.7 g glucose could be produced from 1 kg rice straw with the co-production of 3.84 g FA and 6.98 g *p*-CA after alkaline pretreatment. These results show that the recovery of phenolic compounds from the residual stream could be a green strategy for the low-cost bioconversion of rice straw.

**Electronic supplementary material:**

The online version of this article (10.1007/s12010-018-2751-1) contains supplementary material, which is available to authorized users.

## Introduction

Rice straw is considered to account for the largest portion of available biomass feedstock in the world, and Asia is responsible for 90% of its annual global production [1]. It shows promise as a raw material for the production of biofuels and biomaterials [2]. However, rice straw contains large amounts of lignin and complex polysaccharides, which make it difficult to hydrolyze it to fermentable sugars. Therefore, pretreatment is an essential step in obtaining highly efficient saccharification and fermentation. Thus far, both acid and alkaline pretreatments have been used to improve enzymatic accessibility [3–5]. After pretreatment, the cellulose and hemicellulose in rice straw can be easily hydrolyzed enzymatically into monosaccharides, and thus converted by microorganisms into biofuels and biomaterials [6]. However, the bioconversion of rice straw is very costly. For that reason, a method should be developed for the integrated bioconversion of rice straw to biofuels/biomaterials with high added-value by-products to bring down the overall cost for the bioconversion [7].

Rice straw contains a wide range of phenolic compounds [8, 9]. In our previous study, it was found that phenolic compounds could be released during saccharification of rice straw, and these phenolic compounds exhibited high antioxidant activity [10]. However, they markedly inhibited the growth of lactic acid bacteria and consequently lowered the efficiency of lactic acid production [11]. A similar inhibitory effect on cellulase production by *Trichoderma reesei* has also been found [12]. Therefore, the phenolic compounds need to be removed before saccharification or fermentation to improve the efficiency of the whole process for bioconversion of rice straw. The effect of the recovery of phenolic acids on the cost for ethanol production was discussed, coming the conclusion that the recovery of phenolic acids could significantly reduce the total cost for ethanol production. To lower the inhibitory effect of phenolic acids on yeast growth, an extraction process was needed [13]. Most phenolic acids are ether-linked to lignin and polysaccharides, and the linked phenolic acid could be released by the aids of acid and alkali. During acid or alkaline pretreatments, therefore, phenolic compounds would be released from the rice straw. Commonly, there are two ways to conduct the following saccharification: (1) by first separating the rice straw from the pretreatment liquid, followed by saccharification of the solid fraction, and (2) by saccharification of the rice straw without separation. The latter method could bring about a decrease in fermentation efficiency due to the presence of phenolic compounds and other inhibitors formed during the pretreatments, while the former method could result in the discharge of residual stream, which has a high total organic carbon (TOC) value, containing polysaccharides and polyphenols [14]. In a common viewpoint of bioconversion of lignocellulosic biomass to useful materials, higher temperature and acid/alkaline concentration leads to larger release of inhibitors, resulting in poorer performance of the subsequent process. The common studies on lignocellulosic biomass are focused on how to reduce the release of the inhibitors. Therefore, the former way was considered to be the better way for lowering the cost for bioconversion due to the fewer inhibitors and residual stream. In this study, it was confirmed that the residual stream exhibited high antioxidant activity. If the antioxidants could be produced as by-products during the pretreatment process, the cost of the bioconversion of rice straw into useful materials would be markedly reduced. According to this finding, the former method was considered as an integrated process that could produce antioxidants in the pretreatment step and then monosaccharides in the saccharification step [15]. If the antioxidants could be produced as by-products during the pretreatment process, the cost of the bioconversion of rice straw into useful materials would be markedly reduced.

Although pretreatments of lignocellulosic biomasses have been intensively investigated [16–18], information on the relationship of release of phenolic compounds and saccharification efficiency is scarce. Accordingly, the aims of the present study were to compare the effects of dilute acid or alkaline pretreatment conditions (temperature, time, and concentration) on the release of phenolic compounds and the enzymatic hydrolysis of pretreated rice straw, and consequently on the antioxidant activity of the phenolic compounds in the residual stream, using response surface methodology (RSM). Then, the relationships among the phenolic acids released, enzymatic digestibility of the pretreated rice straw, and antioxidant activities were investigated. Lastly, we explored an integrated pretreatment concept, which aims to simultaneously produce glucose and high-value antioxidants from the residual stream derived from the pretreatment process. To the best of our knowledge, this is the first report on the production of high-value antioxidants as by-products of the pretreatment of rice straw.

## Materials and Methods

### Rice Straw

Rice straw was obtained from several fields on Chongming Island, Shanghai, China. The collected straw samples were air-dried, ground, passed through a 1-mm aperture standard screen, and then kept in an oven at 60 °C prior to pretreatment [18]. National Renewable Energy Laboratory analytical methods [19] were followed to determine the raw material composition in terms of structural carbohydrates. The raw material had the following composition (mg g^−1^): glucose, 342.5 ± 2.5; xylose, 169.5 ± 1.6; arabinose, 34.7 ± 1.2. All chemicals were of analytical grade and obtained from Sinopharm Chemical Reagent Co., Ltd. (Shanghai, China) or Sigma-Aldrich (St. Louis, MO, USA).

### Alkaline Pretreatment

Alkaline pretreatment experiments were performed in 50-mL plastic centrifuge tubes in an autoclave. Dilute NaOH was used for pretreatment of 2 g of ground rice straw at a solid-to-liquid ratio of 1:10 (*w*/*v*). The reaction was carried out in the temperature range of 110–130 °C at different NaOH concentrations (0.5–2.0%, *w*/*v*) and reaction times (10–30 min). After pretreatment, the mixture was centrifuged at 8000*×g* for 5 min. The resulting solids were washed with distilled water until the filtrate registered a neutral pH, and the neutral materials were then combined prior to saccharification. The resulting supernatants were used to analyze the bioactivity and content of phenolic compounds [20]. For the bioactivity analysis, the pH of the supernatants was regulated to neutral with the addition of 4 M HCl. During analysis of the total content of phenolics and phenolic acids, the pH was regulated to 2 by HCl addition, followed by centrifugation at 8000*×g* for 5 min to remove acid-insoluble lignin.

### Acid Pretreatment

The process was similar to the alkaline pretreatment, but dilute H_2_SO_4_ was used for pretreatment under selected conditions (0.5–2.0% H_2_SO_4_, 110–130 °C, 10–30 min, solid-to-liquid ratio of 1:10 *w*/*v*). After pretreatment, the pH of the acid solution was adjusted to 2 with NaOH and the solution was diluted prior to bioactivity and phenolic compound analysis. After pretreatment, the resulting solids were washed with distilled water until the filtrate registered a neutral pH and then used for saccharification.

### Enzymatic Hydrolysis of Pretreated Rice Straw

Enzymatic hydrolysis of pretreated rice straw was carried out in 50-mL conical flasks containing 10% (*w*/*w*) rice straw. *Acremonium* enzyme, a kind of cellulase purchased from Meiji Seika Co. (Japan), in dry solid form was added, and the pH was adjusted to 5. Hydrolysis was performed in a shaker incubator at 45 °C and 200 rpm for 48 h [21]. After hydrolysis, samples were collected and stored prior to analysis of the glucose content. All experiments were performed in triplicate, and averages of data are reported.

### Antioxidant Activity

#### ABTS Radical Cation Inhibition Antioxidant Assay

ABTS solution was prepared by mixing equal portions of potassium persulfate (2.45 mM) and ABTS salt (7 mM). This mixture was kept in the dark for 16 h, and the absorbance was measured at 734 nm. The ABTS solution was diluted with 80% (*v*/*v*) methanol until an absorbance of 0.7 ± 0.005 was obtained. Then, 3.9 mL of this solution were added to 0.1 mL of sample, mixed thoroughly, and kept in the dark for 15 min. The absorbance was measured at 734 nm against a blank (without sample) [22]. A standard calibration curve was prepared using ascorbic acid, and the results were expressed as micromoles of ascorbic acid equivalent per gram of rice straw (μmol AAE g^−1^).

#### Ferric-Reducing Antioxidant Power Assay

The FRAP reagent was freshly prepared by mixing 2.5 mL of 10 mM TPTZ solution in 40 mM HCl with 2.5 mL of 20 mM FeCl_3_ and 25 mL of 0.3 M acetate buffer at pH 3.6. The diluted sample (200 μL) was mixed with 1.8 mL of FRAP reagent, and the absorbance of the reaction mixture was measured at 593 nm after incubation for 10 min [23]. Aqueous standard solutions of ascorbic acid were used to prepare a calibration curve, and the results were expressed in ascorbic acid equivalents (μmol AAE g^−1^).

#### DPPH Radical Scavenging Activity Assay

To determine the DPPH free radical scavenging activity of the samples, 0.1 mL of the diluted sample was mixed with 1 mL of 2,2-diphenyl-1-picryhydrazyl (DPPH) methanol solution (0.2 mM) and allowed to react for 30 min at ambient temperature. The absorbance was measured at 517 nm [24]. Aqueous standard solutions of ascorbic acid were used to prepare a calibration curve, and the DPPH radical scavenging capacity was expressed as micromoles of ascorbic acid equivalent per gram of rice straw (μmol AAE g^−1^).

### Antibacterial Activity of the Supernatants

Gram-positive bacteria *Staphylococcus aureus* ATCC 6538 and gram-negative bacteria *Escherichia coli* ATCC 43894 were chosen to determine the antimicrobial activity of the supernatants. All materials were sterilized in an autoclave at 121 °C for 20 min before the experiments. Every assay was performed in triplicate.

The Oxford cup method was employed to determine the inhibition zone. Firstly, 100 μL of suspension containing 10^7^ CFU mL^−1^ *E. coli* or *S. aureus* was spread on beef extract peptone medium to coat evenly. The medium contained the following (g L^−1^): beef extract 3, peptone 10, NaCl 5. The Oxford cups (6 mm in diameter) were then attached to the corresponding position on the medium. Subsequently, 60 μL aliquots of supernatants were added to the cups, and the plates were incubated at 37 °C for 8 h. Finally, the diameters of the inhibition zones were recorded [25].

### Determination of Total Phenolic Content

Total phenolic content was measured via the Folin–Ciocalteu colorimetric method [26] with minor modification. Approximately 1 mL of the diluted sample (50×) was mixed with 5.5 mL of distilled water and 1.5 mL of Folin phenol solution for 5 min, and then, sodium bicarbonate (10% *w*/*v*) was added along with 2 mL of distilled water. The mixture was stored in the dark at ambient temperature for 4 h. The absorbance of the mixture was recorded using a UV-VIS spectrophotometer (Unico UV-2102C, Shanghai, China) at 765 nm. TP content was quantified based on the standard curve of gallic acid prepared in 80% methanol (*v*/*v*).

### Analysis of Glucose by HPLC

Glucose (Glu) was analyzed by HPLC (LC-20AD, Shimadzu, Kyoto, Japan) using an Aminex HPX-87H column (Bio-Rad, Hercules, CA, USA) at 65 °C. For the mobile phase, 5 mM H_2_SO_4_ was used at a flow rate of 0.6 mL min^−1^.

### Analysis of Phenolic Acids by HPLC

The phenolic acids were analyzed using HPLC (UV detector at 280 nm, EX1600, Exformma, Fairfield, OH, USA) with an Eclipse SDB C18 column (250 mm × 4.6 mm, Agilent, Palo Alto, CA, USA) at 35 °C. Elution was carried out using a linear gradient system consisting of solvent A (water–acetic acid 99.5:0.5) and solvent B (methanol–water–acetic acid 95:0.5:0.5): 0–5 min, 5% B–5% B; 5–10 min, 5% B–25% B; 10–30 min, 25% B–40% B; 30–45 min, 40% B–50% B; 45–55 min, 50% B–100% B; 55–60 min, 100% B–100% B; 60–65 min, 100% B–5% B. All samples were filtered through a 0.45-μm filter before analysis [10].

### Experimental Design and Statistical Analysis

A three-level central composite design analysis with three-level factors was carried out to optimize the levels of *A* (acid or alkaline concentration), *B* (time), and *C* (temperature). Glucose yield and ABTS, FRAP, and DPPH antioxidant activity values as well as TP and phenolic acid yields were taken as the response (*Y*) for the experimental design. The response values for each trial are an average of triplicate measurements. The Design-Expert 8.0 software was used to find the effect of the variables and how they interact. Analysis of variance (ANOVA) was used to determine the significant terms by *p* values. The fit of the models was evaluated by comparing *R*^2^ and adjusted *R*^2^. The relationships between independent variables and response values were analyzed by 3D response surface plots when the third variables were fixed at their central values.

## Results and Discussion

### Influence of Pretreatment Conditions on Glucose Yields

To obtain highly efficient saccharification, both acid and alkaline pretreatments were investigated under the following ranges of pretreatment conditions in this study: 0.5–2.0% (*w*/*v*) H_2_SO_4_/NaOH concentration, 110–130 °C, and 10–30 min reaction time. The response of glucose yield to the varying conditions of acid and alkaline pretreatments was modeled using a response surface. After the pretreatments, the rice straw was filtered from the slurries, followed by saccharification with cellulase (5 FPU g^−1^ straw) at 45 °C for 48 h. The filtered solution was used for assays of antioxidant activity. Cellulase loading has significant effect on glucose yield, and increasing the enzyme loading is a recognized strategy for obtaining a high concentration of glucose. However, the increase in enzyme loading results in the increase in the total cost for bioconversion of rice straw. For that reason, the effect of cellulase loading on the yield of glucose was investigated in the preliminary experiment. As shown in the Fig. S1, in the case of alkaline pretreatment, increased cellulase loading had low effect on saccharification. The glucose concentration did not increase in proportion to the cellulase loading. The increase in enzyme loading from 5 to 40 FPU g^−1^ cellulase increased the glucose yield only by 3.52%. On the other hand, the saccharification of the acid-pretreated rice straw was significantly affected by the enzyme loading. The glucose yield increased in proportion to the cellulase loading. However, the use of a high cellulase concentration is not economically feasible for low-cost production of ethanol. Taken the enzyme cost into account, the cellulase loading was set at 5 FPU g^−1^ straw in this study.

**Fig. 1:**
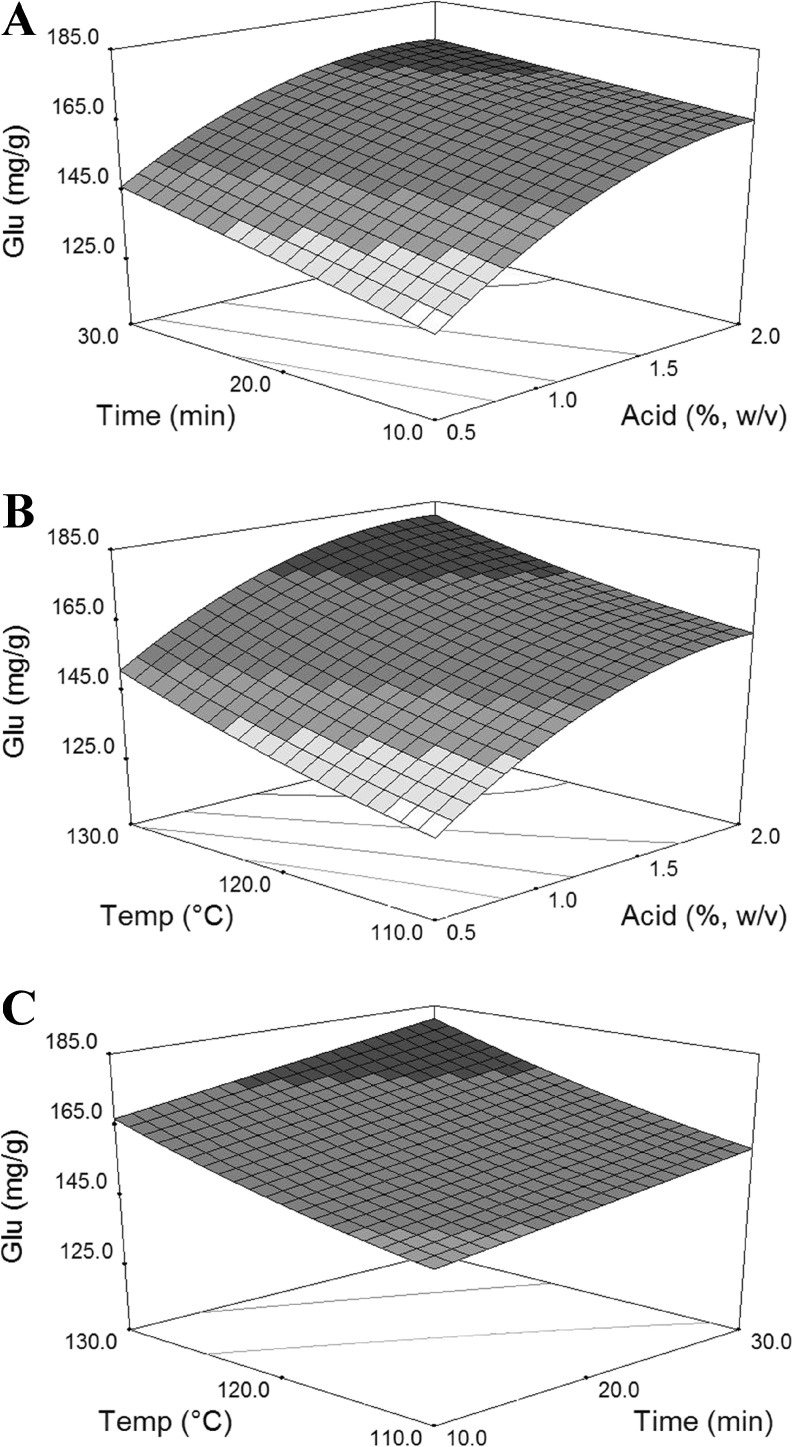
Response surface plots show the effects of independent variables of acid pretreatment on glucose yields. **a** Acid concentration and time. **b** Acid concentration and temperature. **c** Time and temperature

**Fig. 2:**
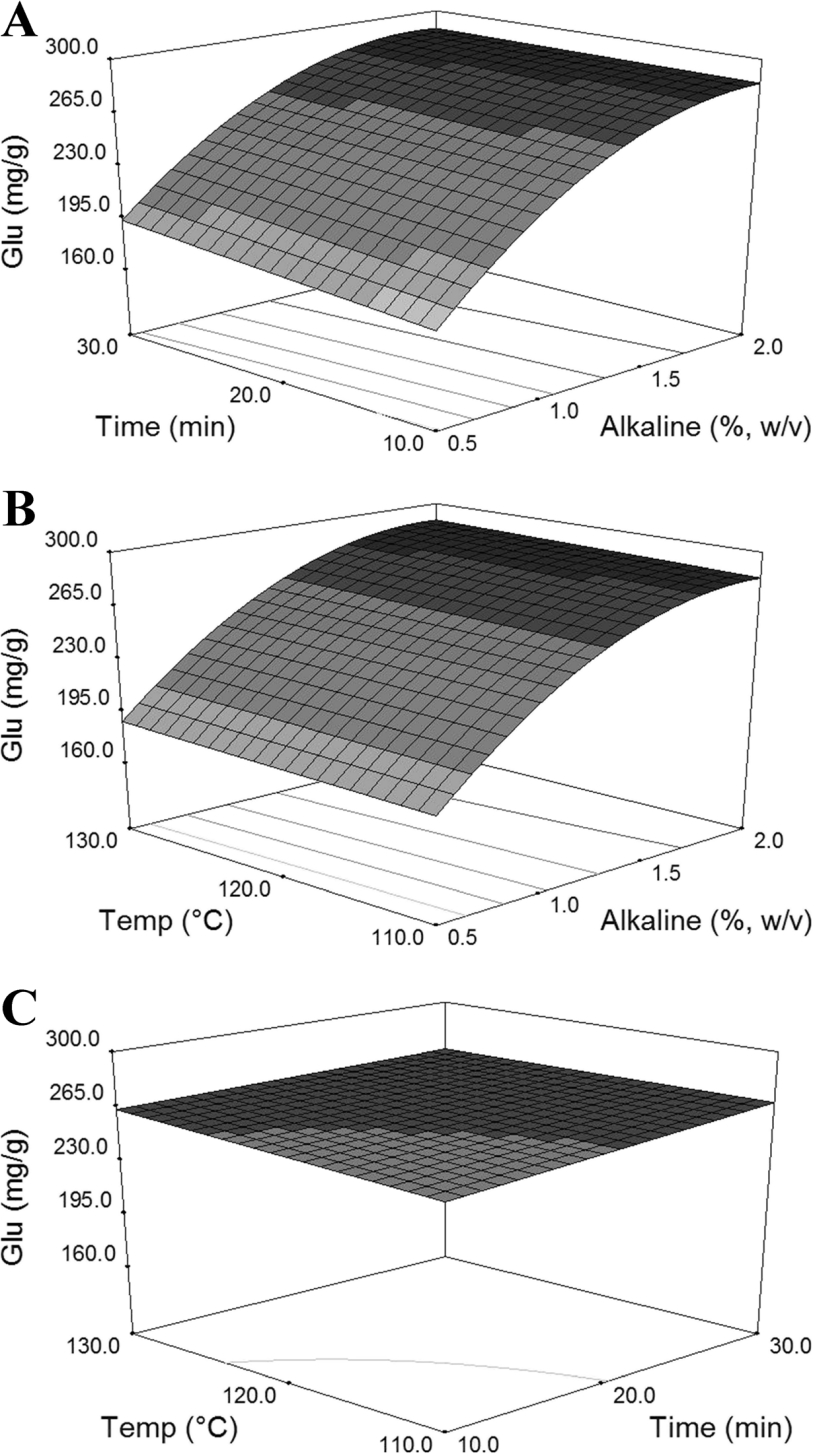
Response surface plots show the effects of independent variables of alkaline pretreatment on glucose yields. **a** Alkaline concentration and time. **b** Alkaline concentration and temperature. **c** Time and temperature

As expected, the chosen ranges of conditions were sufficient to describe the reaction space as evidenced by the broad range of results for the glucose yield (112.6–185.1 mg g^−1^ straw for the acid pretreatment and 112.6–292.6 mg g^−1^ straw for the alkaline pretreatment). The response surface approach was successful in developing an adequate model that describes the glucose yield values. Analysis of variances (ANOVA) of the models for glucose yields are given in Tables S1 and S2. The 3D response surface plots demonstrate the relationships between acid and alkaline pretreatment conditions and response. As shown in Figs. 1 and 2, the acid or alkaline concentration had the most significant effect on the values of glucose yields, which was also demonstrated by the *p* values of the acid or alkaline concentration compared with those of time and temperature (Tables S1 and S2). Under alkaline pretreatment conditions, NaOH concentration was the dominant factor, and the effects of temperature and time on the final yield of glucose were low relative to that of the concentration of NaOH (Fig. 2). Although the concentration of acid played a major role in glucose concentration in the acid pretreatment, temperature and time also significantly affected the glucose concentration (Fig. 1).

To date, there have been many studies on acid and alkaline pretreatments [3, 18]. In this study, when the cellulase loading was set at 5 FPU g^−1^ straw, it was found that the maximum yield of glucose was 85.43% (292.6 mg g^−1^ straw) for the alkaline pretreatment, which was 1.58-fold higher than that for the acid pretreatment, suggesting that the alkaline pretreatment was more efficient for glucose production from rice straw than the acid pretreatment.

### Effects of the Pretreatment Conditions on the Antioxidant Activity of the Supernatants

After pretreatment, the treated rice straw was filtered and the solid was used for enzymatic hydrolysis to produce glucose. The resulting supernatant could be considered to be a kind of industrial residual stream because it contained large amounts of organic matter. As a common gramineous plant, rice straw is similar to Chinese herbal medicinal plants and may also contain some bioactive substances. If we could recover some useful substrates from the supernatant, the residual stream treatment could be considered to be a production process of high-value products. As a result, the cost for the bioconversion of rice straw into useful materials would be markedly reduced.

In order to detect possible biological activity in the supernatant, ABTS, FRAP, and DPPH were selected as antioxidant indicators in this study. As expected, high antioxidant activities were detected in the supernatants under all conditions (Tables 1 and 2). The resulting antioxidant activities of the acid and alkaline supernatants were successfully modeled based upon the pretreatment conditions. Analysis of variances (ANOVA) of the models were given in Tables S3–S8. The significance of each coefficient of the model was determined using the *p* value. A *p* value < 0.05 indicated the significance of independent variables, and a lower *p* value suggested more significant effect. For acid pretreatment, the acid concentration (*A*), time (*B*), and temperature (*C*) all had effects on the antioxidant activities of the supernatants. The terms (such as *B*, *C*, *AB*, *AC*, *BC* for ABTS values, *A*, *B*, *C*, *AC* for FARP values, and *B*, *C* for DPPH values) were significant (*p* < 0.05). In fact, the variations of these terms (such as main terms and interaction ones) can lead to logically effect on the related responses. For alkaline pretreatment, the antioxidant activities of supernatant were affected by the terms of alkaline concentration (*A*), time (*B*), and temperature (*C*). Alkaline concentration (*A*) had the most significant effect on the three antioxidant activities of supernatant, which was proved by the lower *p* value than that of time (*B*) and temperature (*C*). The terms (such as *A*, *B*, *C* for ABTS values, *A*, *C*, *AB* for FARP values, and *A*, *B*, *C*, *AB*, *AC* for DPPH values) are significant (*p* < 0.05). These results showed that the effects of above various factors on the antioxidant activities of the supernatant were not simple linear relationship. Various response surface 3D graphs were generated for ABTS, FRAP, and DPPH antioxidant activities and were shown in Figs. S2 and S3. The trends of the changes in the three kinds of antioxidant activities were similar. The antioxidant activity values increased as the acid concentration, temperature, and time increased, which was similar to the results for the alkaline pretreatment. However, the antioxidant activity is affected by the difference in structure and type and number of substituent on the phenyl ling [27, 28]. The difference in the biomass and the range of environmental factors, testing methods, and pretreatment conditions could result in different antioxidant activity [29]. Akpinar et al. reported that the alkaline hydrolysates of wheat straw showed 13.70 μmol TE (trolox equivalent) g^−1^ ABTS and 3.20 μmol TE g^−1^ DPPH, whereas the acid hydrolysis liquors of wheat straw (120 °C, 4% H_2_SO_4_, 30 min) showed 1.60 μmol TE g^−1^ ABTS and 1.30 μmol TE g^−1^ FRAP activities [30]. These results indicate that alkaline treatment exhibited much higher performance than acid treatment in the formation of phenolic compounds with high antioxidant activity, which was in agreement with our results.

**Table 1:** Experimental design and antioxidant activity results for the acid pretreatment supernatants of rice straw

Run	A	B	C	ABTS	FRAP	DPPH
	Acid (%, *w*/*v*)	Time (min)	Temp (°C)	(μmol AAE g^−1^ straw)		
1	0.5	10	110	19.85	8.88	5.72
2	2.0	10	110	46.07	20.63	9.22
3	0.5	30	110	25.50	12.81	10.35
4	2.0	30	110	49.53	35.57	18.21
5	0.5	10	130	28.53	15.05	8.36
6	2.0	10	130	48.60	35.61	17.38
7	0.5	30	130	44.87	35.71	18.31
8	2.0	30	130	55.09	41.36	19.77
9	1.25	20	120	49.10	40.36	19.33
10	1.25	20	120	46.27	41.10	19.09
11	1.25	20	120	48.48	39.80	19.29
12	1.25	20	120	49.83	42.50	18.78
13	1.25	20	120	47.33	40.90	19.61
14	1.25	20	120	48.30	42.10	18.90
15	0.0	20	120	29.09	9.31	10.78
16	1.25	20	105	45.42	22.03	11.90
17	1.25	20	136	53.08	48.19	22.56
18	1.25	5	120	40.64	18.24	9.96
19	1.25	37	120	52.68	44.25	21.22
20	2.51	5	120	36.81	31.06	12.98

**Table 2:** Experimental design and antioxidant activity results for the alkaline pretreatment supernatants of rice straw

Run	A	B	C	ABTS	FRAP	DPPH
	Alkaline (%, *w*/*v*)	Time (min)	Temp (°C)	(μmol AAE g^−1^ Straw)		
1	0.5	10	110	32.00	39.17	21.24
2	2.0	10	110	53.03	51.61	34.20
3	0.5	30	110	34.85	40.33	21.79
4	2.0	30	110	63.69	66.72	39.82
5	0.5	10	130	35.41	43.25	23.17
6	2.0	10	130	64.60	47.62	42.30
7	0.5	30	130	41.20	48.92	25.37
8	2.0	30	130	67.85	67.27	45.67
9	1.25	20	120	55.22	52.52	32.85
10	1.25	20	120	55.03	52.93	32.55
11	1.25	20	120	53.96	55.81	32.94
12	1.25	20	120	52.93	49.47	32.40
13	1.25	20	120	53.12	51.51	33.80
14	1.25	20	120	52.90	52.81	33.86
15	0.0	20	120	29.09	9.31	10.78
16	1.25	20	105	54.15	45.48	31.79
17	1.25	20	136	60.50	54.54	38.17
18	1.25	5	120	50.49	46.00	32.04
19	1.25	37	120	57.91	52.96	35.96
20	2.51	5	120	58.88	51.92	38.42

### Relationships Among Phenolic Compounds, Antioxidant Activities, and Saccharification Efficiency

In this study, it was found that the supernatant exhibited high antioxidant activities, which were significantly correlated with the pretreatment conditions. In our previous study, more than 10 kinds of phenolic acids in rice straw were detected [12] and their antioxidant activity was confirmed [10]. Because the phenolic acids esterified to polysaccharides would be released by cleavage of the ester linkages in the lignin–polysaccharide complexes under acid or alkaline conditions, it is feasible to combine the production of phenolic acids with that of monosaccharides by acid or alkaline pretreatment. Therefore, the supernatants exhibiting the highest antioxidant activities obtained in the acid and alkaline pretreatments were selected to determine the contents of total polyphenols by the Folin–Ciocalteu colorimetric method and phenolic acids by HPLC. The Folin–Ciocalteu colorimetric method showed large amounts of polyphenols in the supernatants, and moreover, chlorogenic acid, vanillic acid, caffeic acid, *p*-coumaric acid, vanillin, ferulic acid, and meson acid were detected by the HPLC method (Table 3). FA and *p*-CA accounted for about 84% of the content of total phenolic acids.

**Table 3:** Compositions of the total phenolics and phenolic acids in the acid/alkaline supernatants of rice straw

Compound	Concentration (mg g^−1^ straw)	
	Acid supernatant	Alkaline supernatant
Total phenolic	10.34 ± 0.58	26.92 ± 0.71
Gallic acid	0.03 ± 0.00	0.02 ± 0.00
Chlorogenic acid	0.02 ± 0.00	0.03 ± 0.00
Phthalic acid	0.06 ± 0.01	0.04 ± 0.00
Vanillic acid	0.09 ± 0.00	0.57 ± 0.01
Caffeic acid	0.04 ± 0.00	0.16 ± 0.01
Vanillin	0.13 ± 0.00	0.19 ± 0.00
*p*-Courmaric acid	0.40 ± 0.05	7.08 ± 0.03
Ferulic acid	0.84 ± 0.03	3.91 ± 0.07
Sinapic acid	0.07 ± 0.01	0.48 ± 0.00

Acid supernatant: 2% H_2_SO_4_, 130 °C, 30 min. Alkaline supernatant: 2% NaOH, 130 °C, 30 min

**Fig. 3:**
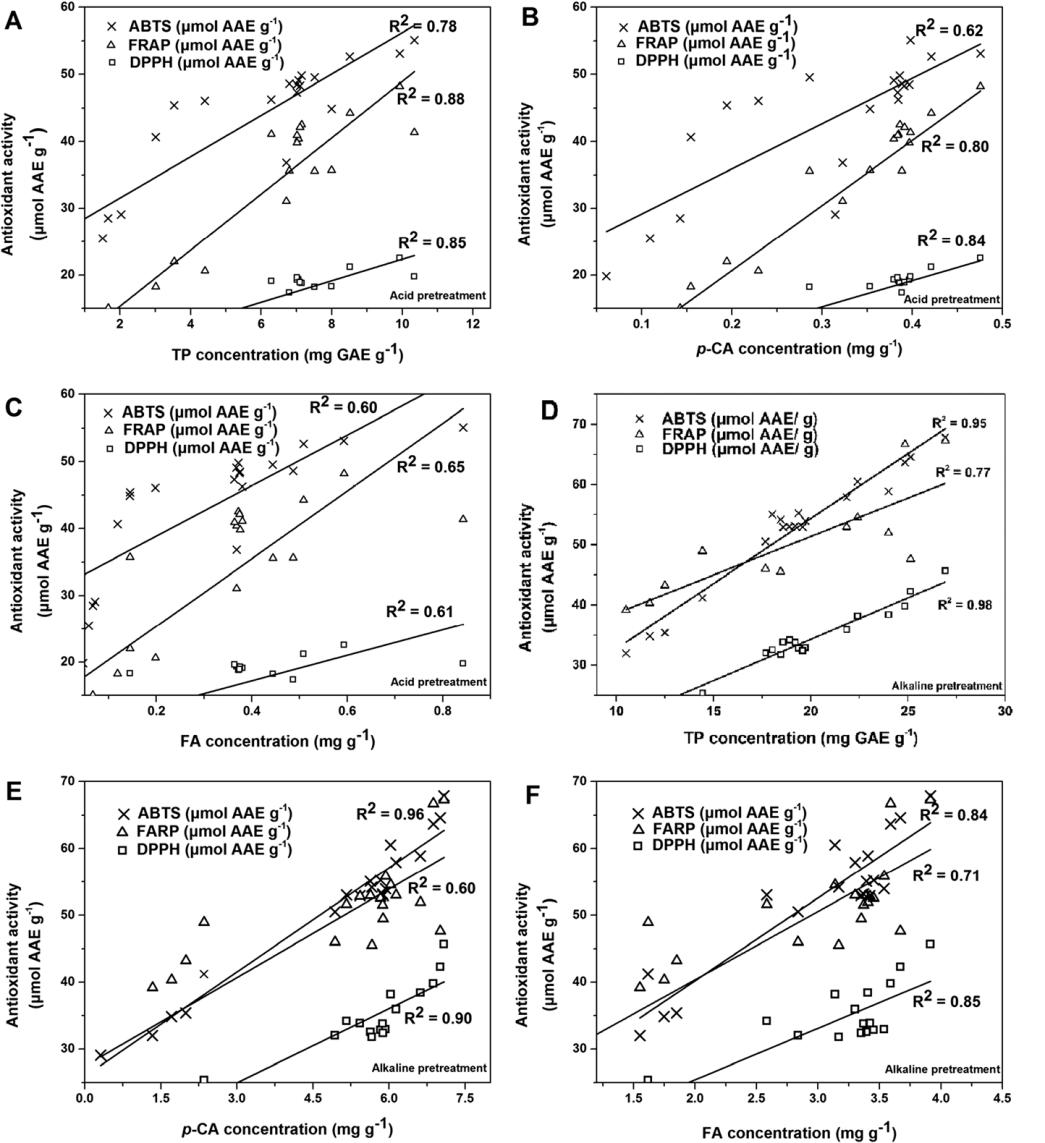
Correlation between TP, *p*-CA, and FA and total antioxidant activity for all acid/alkaline supernatants after pretreatment. **a**–**c** Acid pretreatment results. **d**–**f** Alkaline pretreatment results

In order to prove that the phenolic compounds were the main factors affecting the antioxidant activities, the antioxidant activities of the supernatants measured by ABTS, FRAP, and DPPH were plotted against the TP, *p*-CA, and FA contents (Fig. 3). For the alkaline pretreatment, the contents of TP, *p*-CA, and FA were highly correlated with the activities of ABTS and DPPH (*R*^2^ > 0.80), but were less well correlated with the activity of FRAP (*R*^2^ < 0.80). On the other hand, much lower correlations for the acid pretreatment were found between the contents of TP, *p*-CA, FA, and the activity of ABTS (*R*^2^ < 0.79), and the correlation between the FA content and ABTS activity was especially low (*R*^2^ < 0.61). This could be due to a reduction in the contents of phenolic compounds as a result of the acid pretreatment. Another cause could be the presence of polysaccharides in the extracts. Xu et al. [31] reported that water-soluble polysaccharides from *Pteridium aquilinum* had strong FRAP activity and moderate DPPH activity. Therefore, in the future, purification of the phenolic compounds needs to be performed to investigate this correlation in detail.

**Fig. 4:**
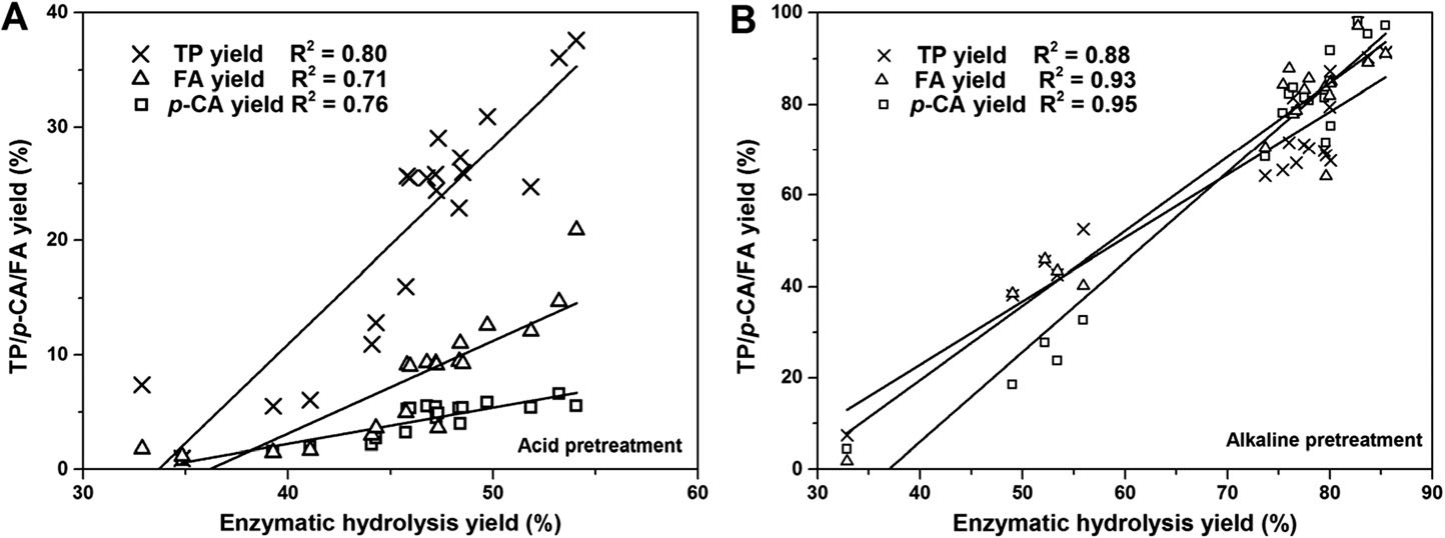
Correlation between TP, *p*-CA, and FA yields and enzymatic hydrolysis yield. **a** The correlation between TP, *p*-CA, and FA release and enzymatic hydrolysis for the acid pretreatment. **b** The correlation between TP, *p*-CA, and FA release and enzymatic hydrolysis for the alkaline pretreatment

In addition, there were good linear correlations between the contents of phenolic compounds and saccharification efficiency (*R*^2^ > 0.70) (Fig. 4). The *R*^2^ values between TP content, the release of *p*-CA and FA, and the yield of glucose were 0.80, 0.76, and 0.71, respectively, for the acid pretreatment, while *R*^2^ = 0.88, 0.95, and 0.93, respectively, were observed for the alkaline pretreatment. These results indicate that the release of phenolic compounds could be correlated with the destruction of polysaccharides.

Above all, it is obvious that there were correlations among the release of phenolic compounds, the antioxidant activity of the supernatants, and saccharification efficiency, suggesting that phenolic compounds were the main factors affecting the antioxidant activities and that they could be produced efficiently without any decrease in saccharification efficiency. To retain the bioactivity of phenolic compounds, losses of these bioactive compounds during pretreatments should be minimized. The stability of the phenolic acids was studied in the temperature range 110–130 °C and acid/alkaline concentration of 0.5–2.0% (Fig. S4). For both the phenolic acids, significant increase was visible with the increasing of temperature and acid/alkaline concentration, which should be attributed to an increased extractability of phenolic acids under the condition of the pretreatments. Based on the data, the temperature-acid/alkaline concentration could be valuable for the production of phenolic acids. This finding could give us a new insight into the development of optimum conditions for the pretreatment of rice straw.

### Antimicrobial Activities of the Phenolic Compounds

Many plants have been found to yield compounds having antimicrobial properties. Inhibitory effects of curcumin phenolic compounds isolated from the plant *Curcuma longa* on bacteria have also been observed [32]. In our previous study, it was confirmed that phenolic compounds released during saccharification exhibited inhibitory effects on the growth of microoganisms [11, 12]. The amounts of TP, especially FA and *p*-CA, obtained in the alkaline pretreatment were much higher than those with enzymes, which had been reported previously [10]. Considering the production of phenolic compounds alone, they are commonly recovered from straws under mild alkaline conditions [33]. For a better yield of glucose, however, the pretreatment of rice straw was carried out under severe conditions in this study. Some useful substrates would be destroyed under such conditions. Therefore, it was necessary to confirm whether the phenolic compounds released under severe conditions exhibit antimicrobial activity.

Two microorganisms, *E. coli* and *S. aureus*, were selected to test the ability of the phenolic compounds to act as antimicrobial agents [34], and the Oxford cup diffusion method was used to determine the antibacterial properties of the phenolic compounds against the two microorganisms at different temperatures, concentrations of NaOH, and reaction times (Table 4). For reference, the pretreatment was also carried out under low temperature, low concentration of NaOH, and short reaction time conditions. The zone of inhibition results confirmed that the phenolic compounds exhibited antibacterial activity against the two organisms, but this was found to be more pronounced against *E. coli*. Higher temperature, higher concentration of NaOH, and longer reaction time resulted in a greater antimicrobial effect, suggesting that it is possible to produce phenolic compounds as antimicrobial agents under severe conditions. Indeed, the severe conditions reduced the specific antimicrobial effects of the phenolic compounds. However, milder conditions resulted in poor inhibitory effects due to the lower contents of phenolic compounds. As a result, the conditions of temperature < 100 °C, concentration of NaOH < 2%, and time < 20 min seemed not to be suitable for the production of antimicrobial agents from rice straw. According to the results, it has been demonstrated that the phenolic compounds of rice straw obtained under severe alkaline conditions have potential antimicrobial effects.

**Table 4:** Inhibition results for the different alkaline pretreatment supernatants

Run	Alkaline (%, *w*/*v*)	Temp (°C)	Time (min)	TP (mg GAE g^−1^ straw)	Inhibition zone (cm)	
					*E. coli*	*S. aureus*
1	2.0	60	20	7.49 ± 0.00	0.60 ± 0.00	0.60 ± 0.00
2	2.0	70	20	7.52 ± 0.18	0.60 ± 0.00	0.60 ± 0.00
3	2.0	80	20	5.59 ± 0.73	0.60 ± 0.00	0.60 ± 0.00
4	2.0	90	20	8.11 ± 0.21	0.60 ± 0.00	0.60 ± 0.00
5	2.0	100	20	9.82 ± 0.43	0.60 ± 0.00	0.60 ± 0.00
6	0.5	110	10	10.50 ± 0.25	0.60 ± 0.00	1.31 ± 0.02
7	2.0	110	10	18.91 ± 0.30	1.32 ± 0.01	1.36 ± 0.03
8	2.0	110	20	19.37 ± 0.33	1.65 ± 0.03	1.40 ± 0.06
9	2.0	110	30	24.85 ± 0.63	1.14 ± 0.11	1.10 ± 0.07
10	2.5	110	10	26.15 ± 0.42	1.43 ± 0.01	1.40 ± 0.06
11	2.0	120	20	21.98 ± 0.51	1.73 ± 0.06	1.53 ± 0.05

### Significance of an Integrated Process for Co-Production of Phenolic Compounds and Glucose from Rice Straw

**Fig. 5:**
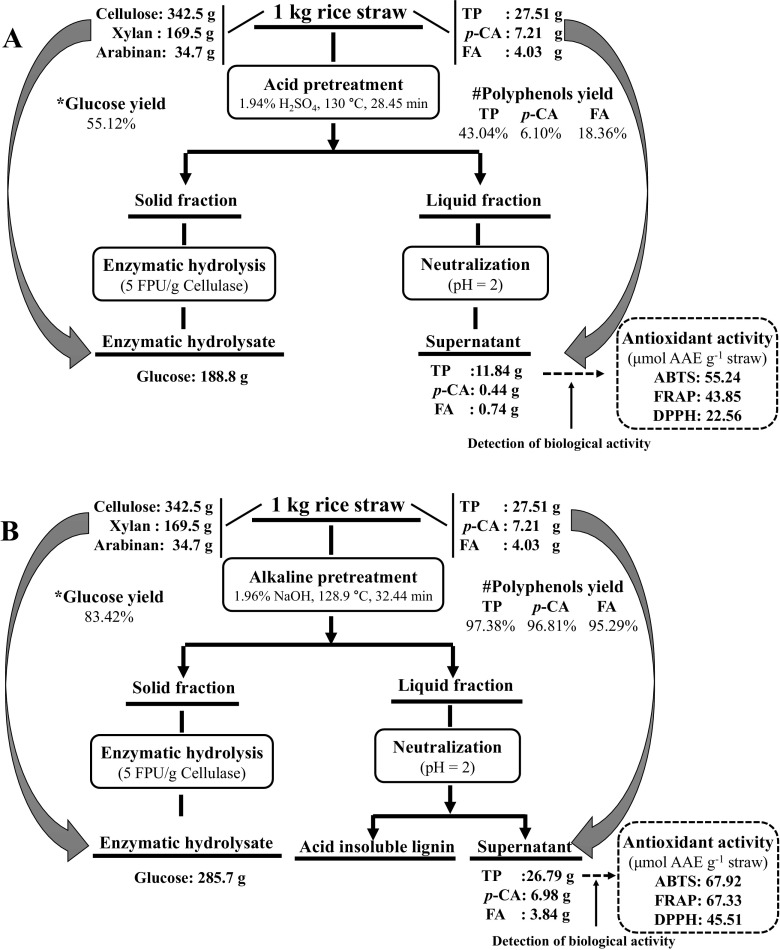
Overall mass balance for the processes for co-production of phenolic compounds and glucose. **a** Acid pretreatment. **b** Alkaline pretreatment

Acid and alkaline pretreatments are commonly used for the bioconversion of rice straw into biofuels and biomaterials [16]. To discuss the significance of co-production of glucose and phenolic compounds, the pretreatment processes were optimized by maximizing the desirability of all the responses. Optimum conditions of the alkaline pretreatment were found to be 1.96% NaOH (%, *w*/*v*), 32.44 min, and 128.9 °C, while optimum conditions of the acid pretreatment were 1.94% H_2_SO_4_ (%, *w*/*v*), 28.45 min, and 130 °C. Under these conditions, the experimental values were in agreement with the predicted values (Table S9), and the glucose yields, antioxidant activities, and phenolic compound contents of the supernatants are given in Fig. 5. Comparing the experimental values, the optimal alkaline pretreatment conditions gave approximately 1.51-fold higher glucose yield, 1.22-fold higher ABTS activity, 1.39-fold higher FRAP activity, and 2.03-fold higher DPPH activity than the acid pretreatment. As for the phenolic acids in the supernatants, the alkaline-based extract had approximately 2.26-fold higher TP contents, 15.19-fold higher *p*-CA content, and 4.59-fold higher FA content compared to the acid pretreatment.

The antioxidant and antibacterial effects of the phenolic compounds render them effective as food preservatives. However, it should be noted that phenolic compounds obtained via chemical methods would have fewer applications in the food and pharmaceutical industries than those obtained via biological methods. Therefore, the possibility of the use of phenolic compounds as antimicrobial agents was investigated [35, 36]. Moreover, phenolic compounds could be used as biological pesticides because they could be produced in large quantities due to the huge amounts of available rice straw. According to the Food and Agriculture Organization, from 600 to 900 million tons of rice straw are produced globally every year. Under the optimum conditions of the alkaline pretreatment, 285.7 kg of glucose could be produced from 1 t of rice straw with the release of 26.79 kg of TP containing 3.84 kg of FA and 6.98 kg of *p*-CA (Fig. 5). In addition, the supernatant from 1 t of rice straw would exhibit 67.92, 67.33, and 45.51 mol equivalent of ascorbic acid in ABTS, FRAP, and DPPH antioxidant activities. The large amount of phenolic compounds derived from rice straw would markedly reduce the cost of the biological pesticide, and consequently reduce the demand for chemical pesticides, leading to green sustainable production of crops.

The results obtained in this study demonstrate that the phenolic compounds derived from rice straw exhibit high inhibitory effects on the growth of pathogens, giving us a new insight into the application of phenolic compounds as antimicrobial agents. These phenolic compounds could be separated and purified by chromatographic methods or adsorption. The production of phenolic compounds could be considered to be a strategy for lowering the total cost of the bioconversion of rice straw, and the resulting residual stream could be easily treated via an activated sludge process due to the decrease in total organic carbon value and antimicrobial activity. In future studies, the inhibitory effects of phenolic compounds on plant pathogens should be investigated in detail.

## Conclusion

In this study, it was confirmed that the residual stream of acid and alkaline pretreatments of rice straw exhibited high antioxidant activities. Response surface methodology was successful in developing an adequate model that describes the enzymatic saccharification and the release and antioxidant activities of the phenolic compounds. Acid and alkaline concentrations were demonstrated to be the most significant parameters affecting all response values. The antioxidant activities were strongly linearly correlated with the contents of phenolic compounds and the enzymatic hydrolysis yield of glucose. The phenolic compounds exhibited inhibitory effects on pathogens, suggesting their potential as biological pesticides. The co-production of antimicrobial agents and biofuels/biomaterials could enable expansion of the scope of applications of rice straw with good prospects for enhanced applications.

## Electronic supplementary material

ESM 1 (DOCX 1159 kb)
