# Supplementary material for: Tapping the Bioactivity Potential of Residual Stream from Its Pretreatments May Be a Green Strategy for Low-Cost Bioconversion of Rice Straw
Source: Appl Biochem Biotechnol. 2018 Apr 16;186(3):507–24. doi: 10.1007/s12010-018-2751-1 (PMC6209036; doi:10.1007/s12010-018-2751-1)
Supplement: Supplementary file 1 — (DOCX 1159 kb) [file 12010_2018_2751_MOESM1_ESM.docx]

**Tapping the bioactivity potential of residual stream from its pretreatments may be a green strategy for the low-cost bioconversion of rice straw**

Xingxuan Chen, Xiahui Wang, Yiyun Xue, Tian-Ao Zhang, Jiajun Hu, Yiu Fai Tsang, Min-Tian Gao^*^

**Supplementary materials**

**Fig. S1.** Effect of cellulase loading on glucose yield.

**Fig. S2.** Response surface plots shows the effect of independent variables of acid hydrolysates on ABTS, FRAP and DPPH antioxidant activities.

**Fig. S3.** Response surface plots shows the effect of independent variables of alkaline hydrolysates on ABTS, FRAP and DPPH antioxidant activities.

**Fig. S4.** Temperature and acid/alkaline stability of phenolic acids treated for 30 min. (A): acid pretreatment; (B): alkaline pretreatment

**Table S1-S8.** The results of ANOVA analysis for the responses of acid and alkaline pretreatment

**Table S9.** Experimental data of the validation of predicted values at optimal pretreatment conditions


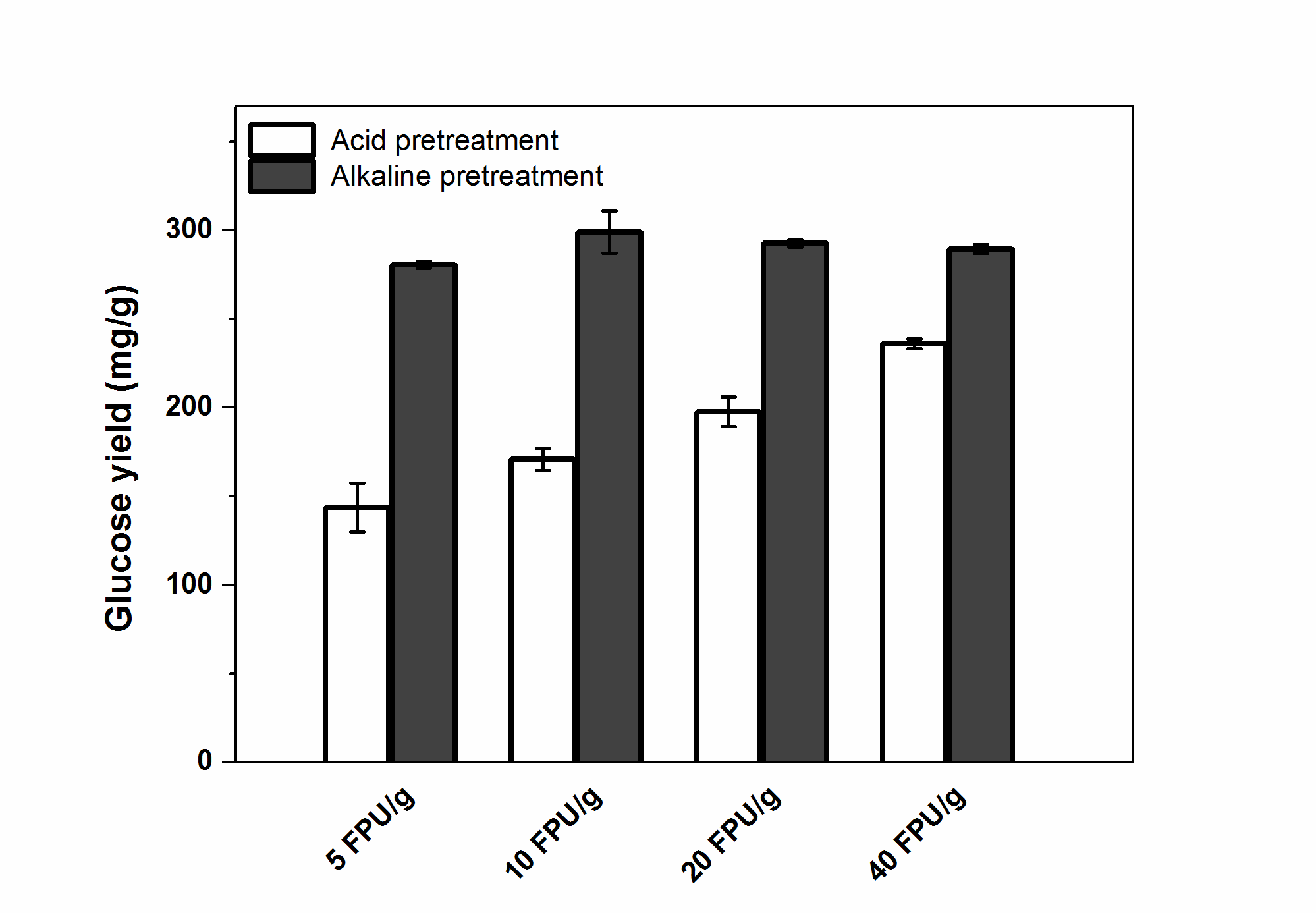


**Fig S1.**


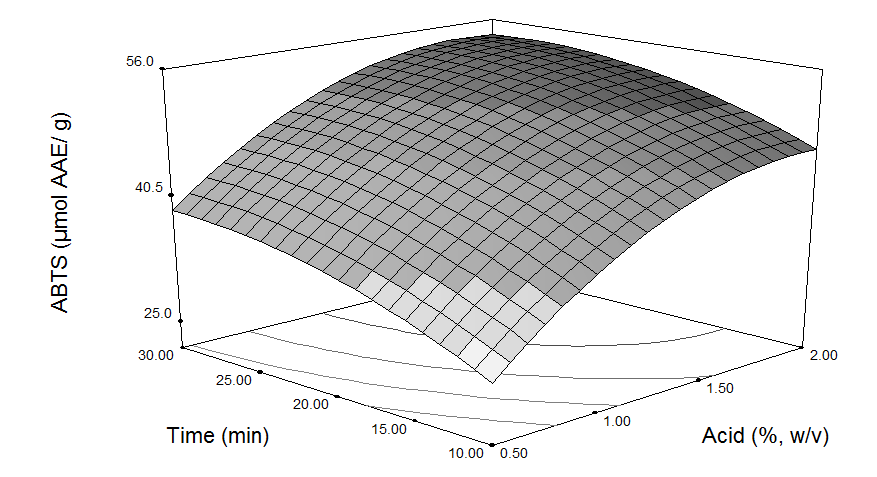

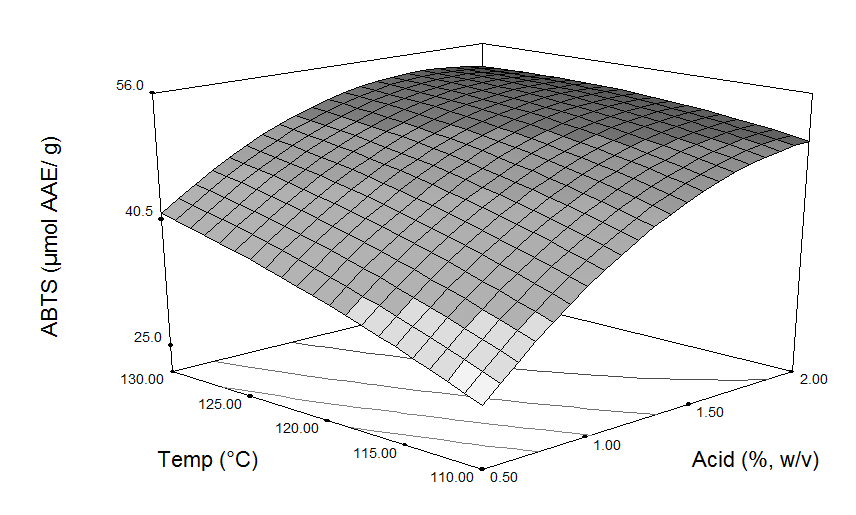

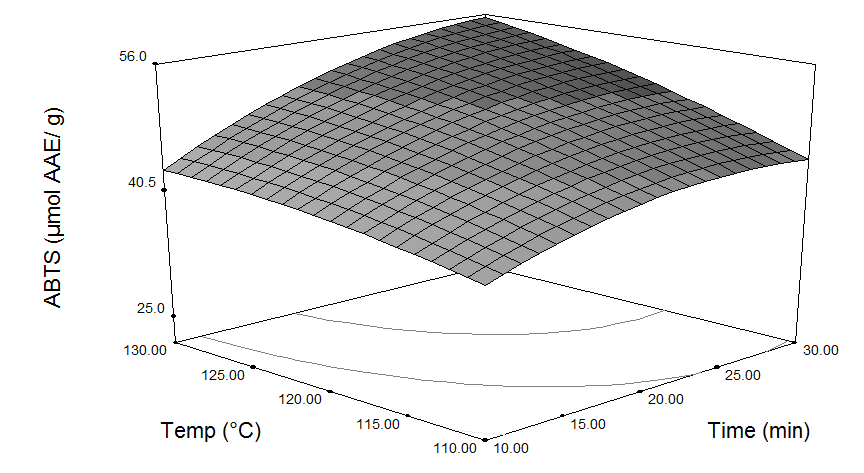


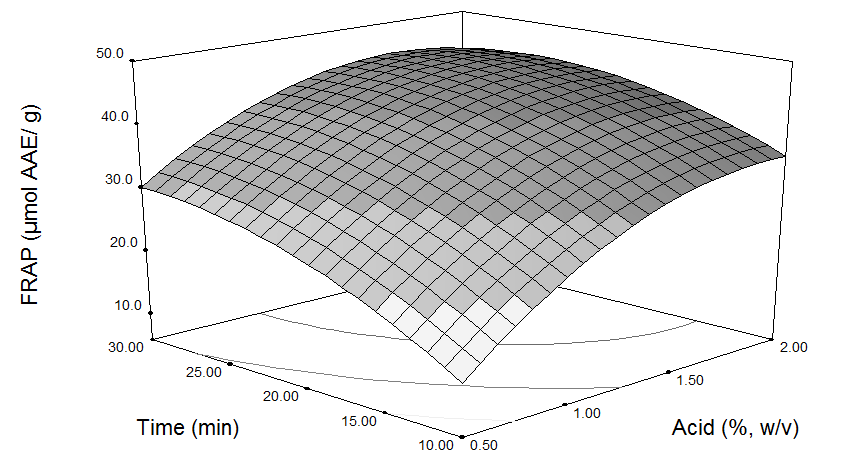

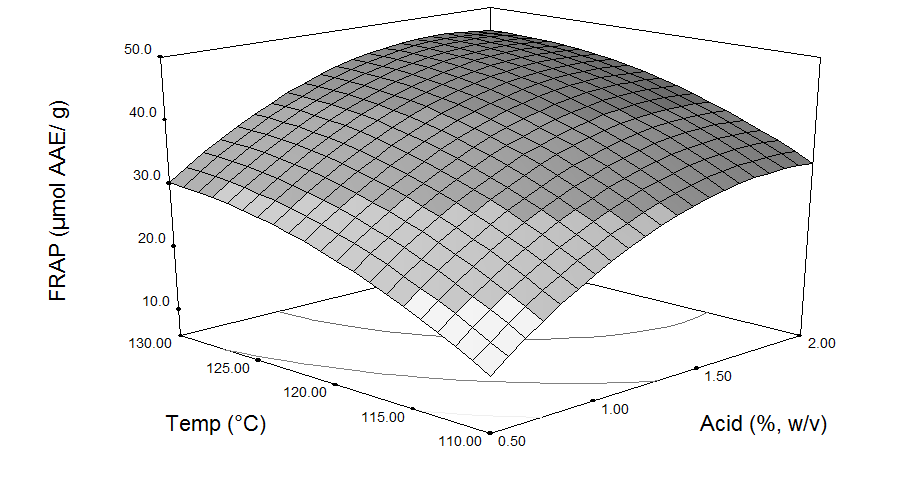

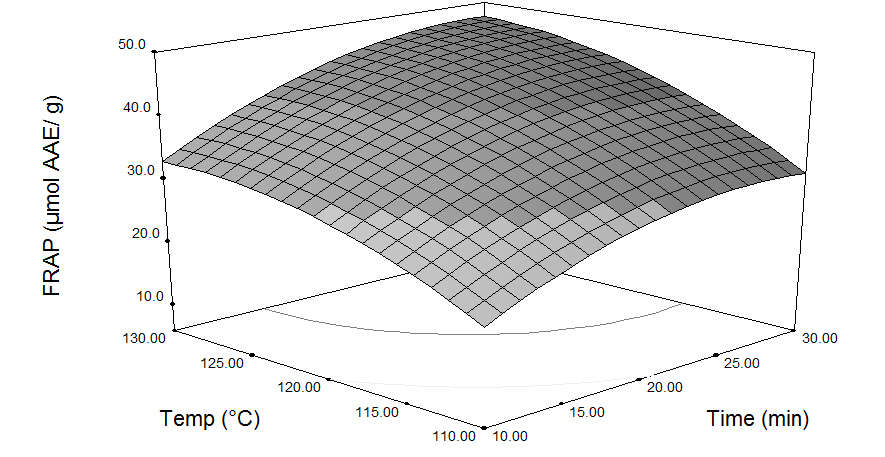


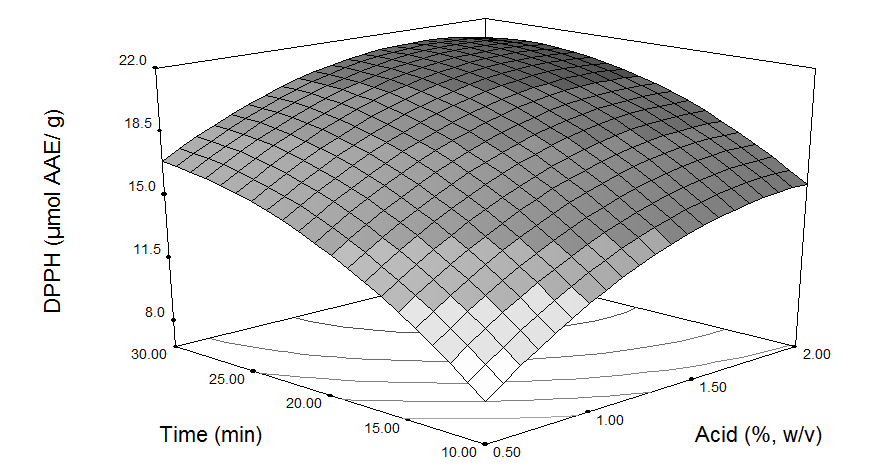

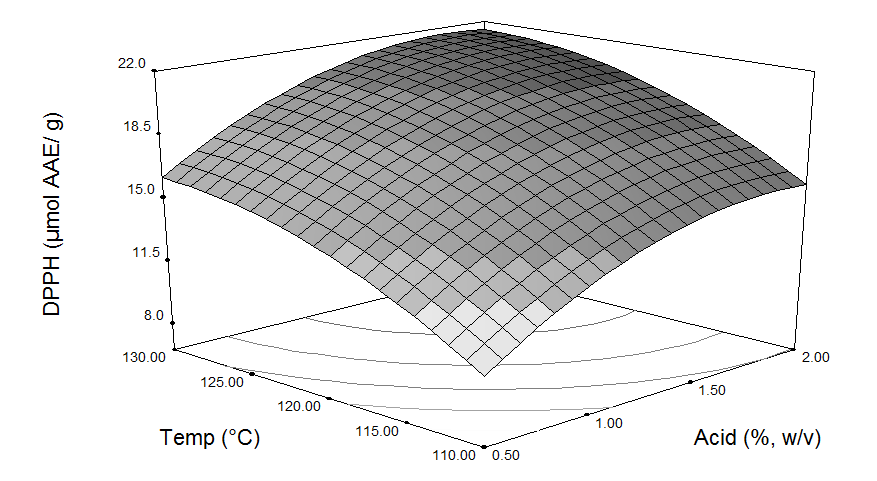


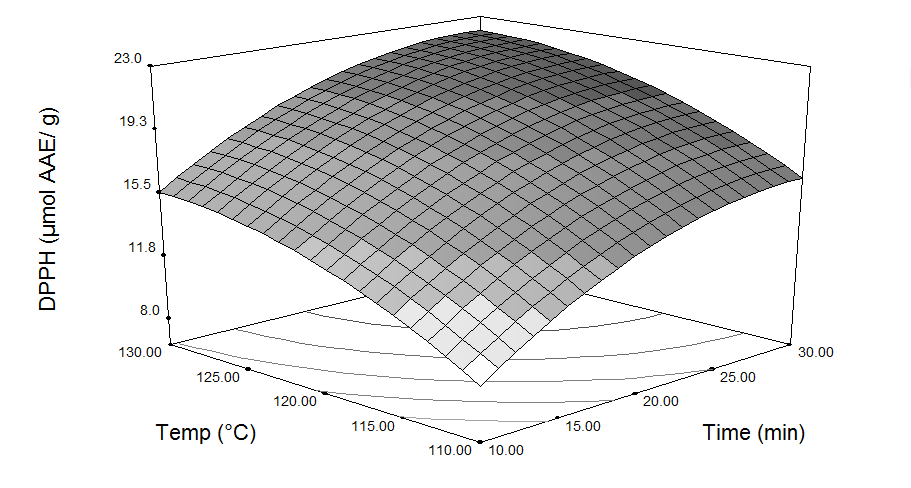


**Fig. S2.**


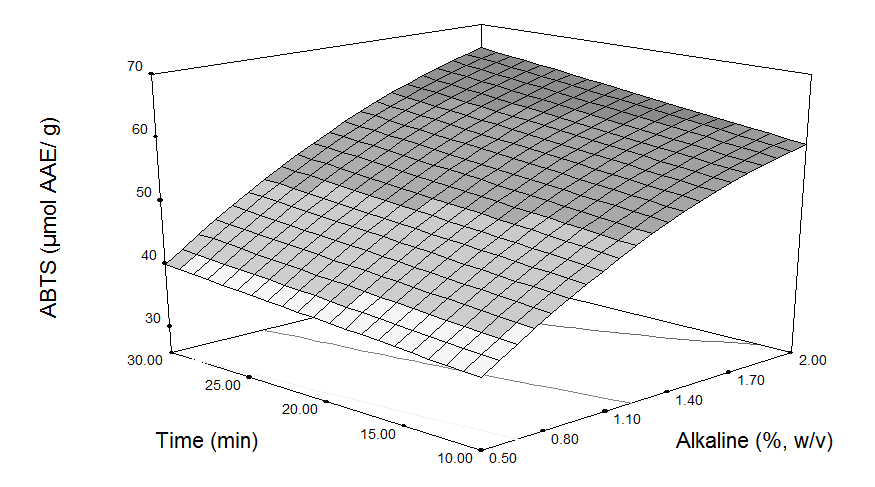

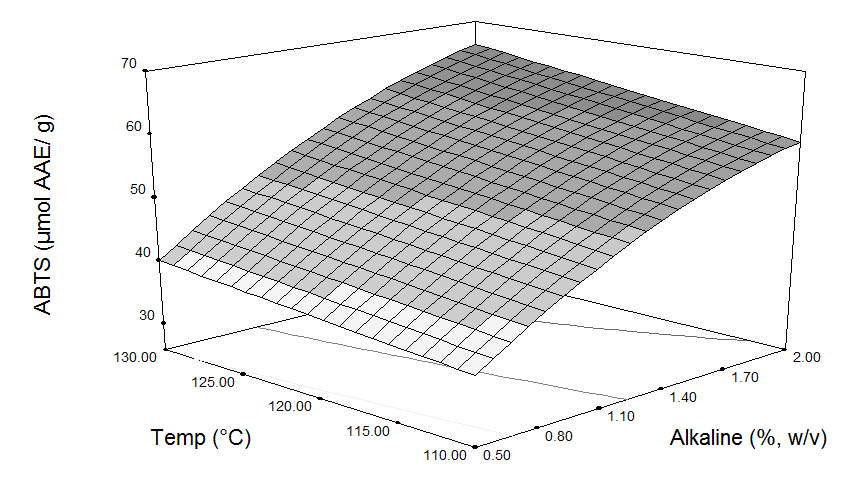

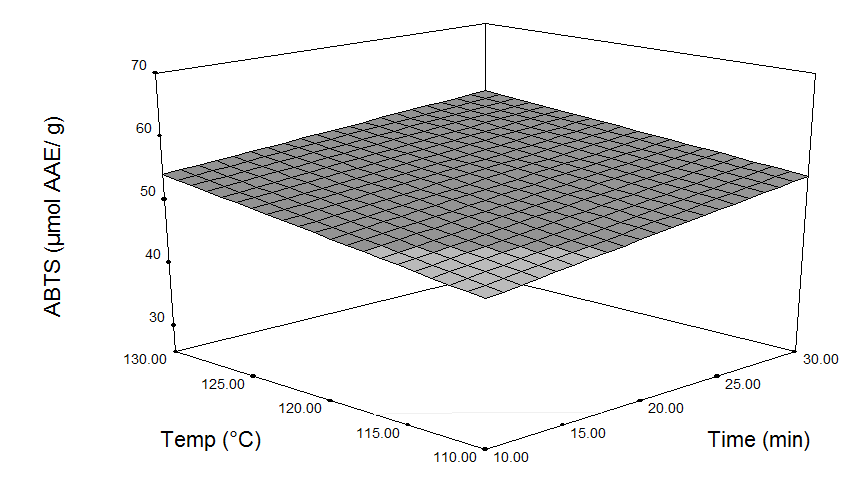


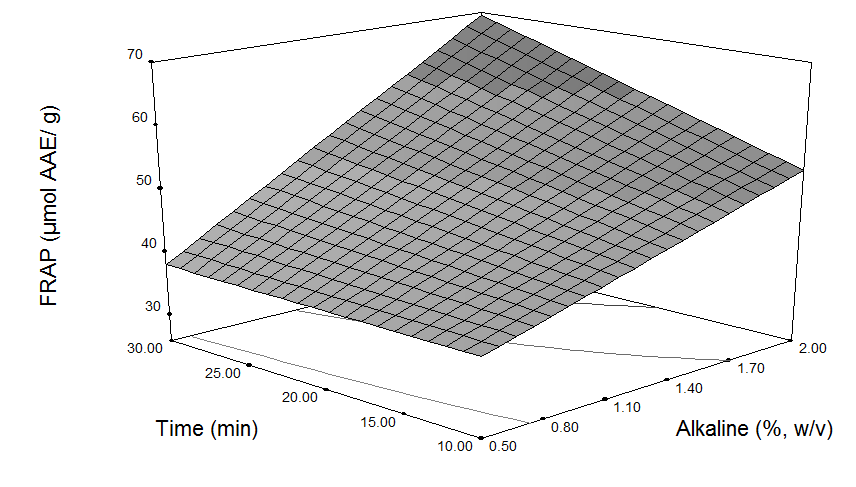

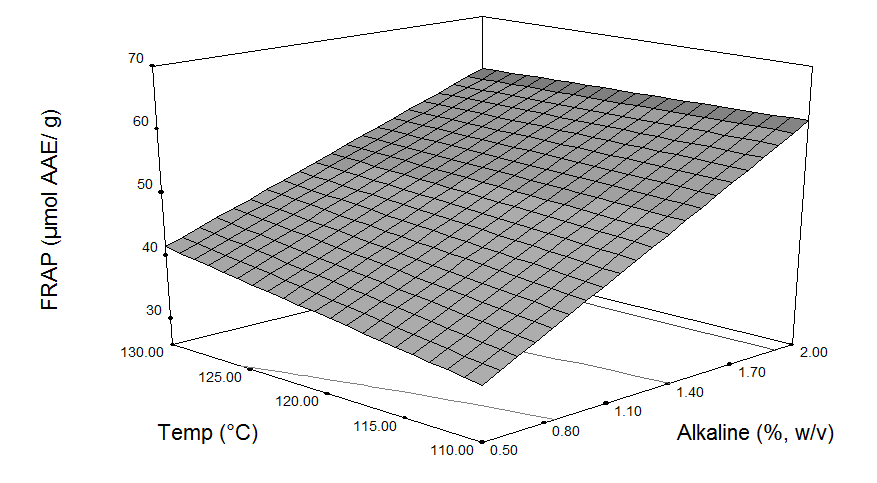

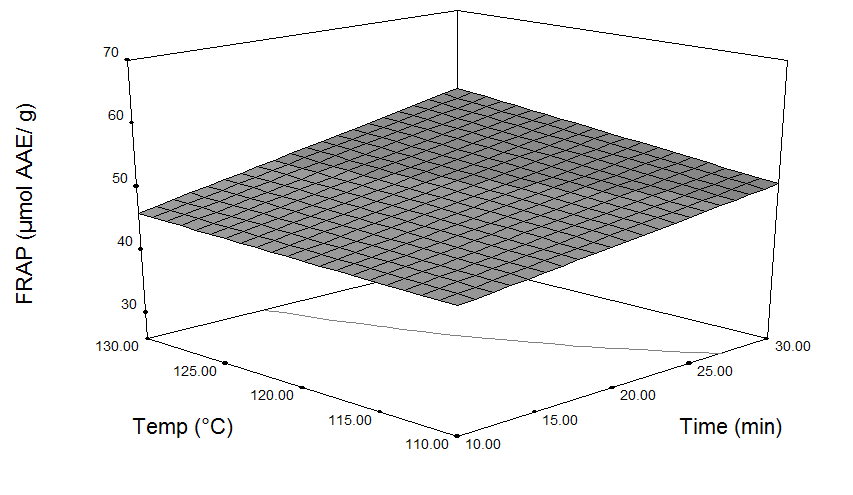


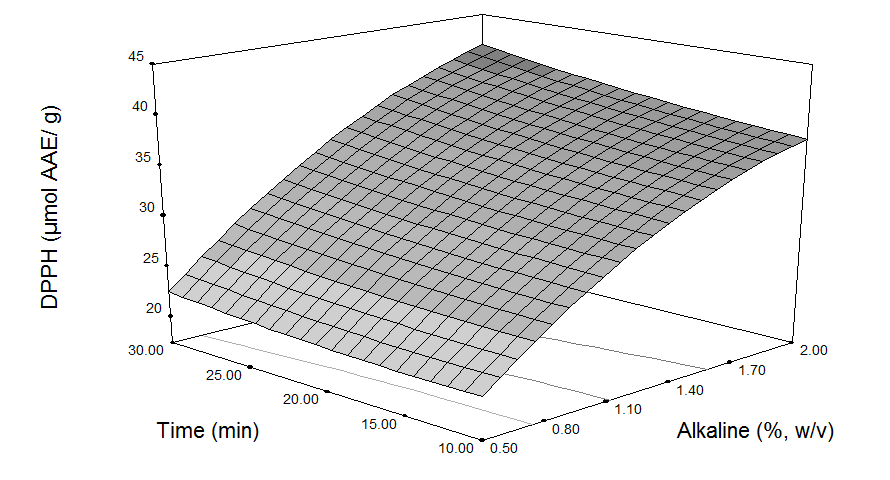

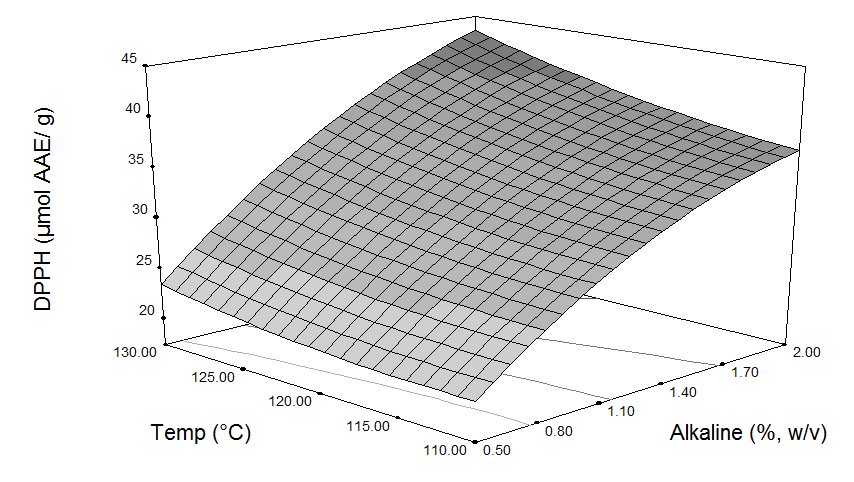

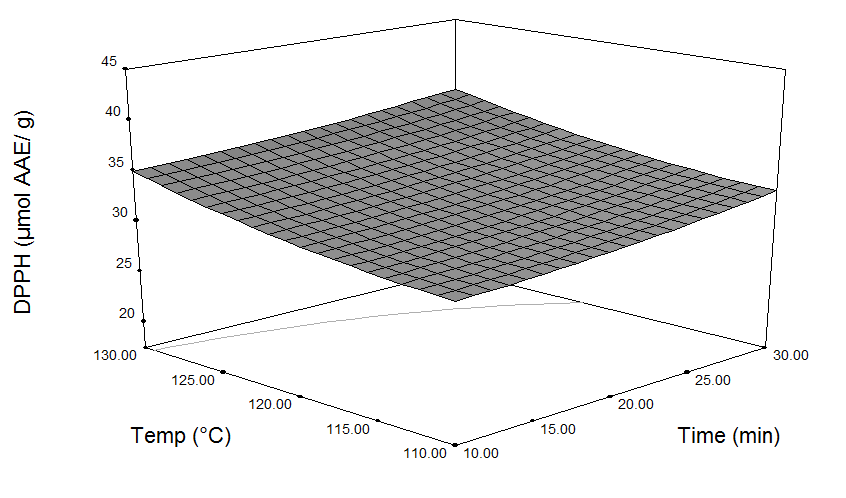


**Fig. S3.**







**Fig. S4.**

**Table S1-S8 The results of ANOVA analysis for the responses of acid and alkaline pretreatment**

| **Table S1**: The results of ANOVA analysis for the response of glucose yield of acid pretreatment   \|  \| Sum of \| Mean \| F \| p-value \| \| --- \| --- \| --- \| --- \| --- \| \| Source \| Squares \| Square \| Value \|  \| \| Model \| 6531.11 \| 725.68 \| 63.91 \| < 0.0001 \| \| A-Acid \| 2930.50 \| 2930.50 \| 258.10 \| < 0.0001 \| \| B-Time \| 573.12 \| 573.12 \| 50.48 \| < 0.0001 \| \| C-Temp \| 1437.36 \| 1437.36 \| 126.59 \| < 0.0001 \| \| AB \| 65.89 \| 65.89 \| 5.80 \| 0.0367 \| \| AC \| 9.46 \| 9.46 \| 0.83 \| 0.3828 \| \| BC \| 2.76 \| 2.76 \| 0.24 \| 0.6326 \| \| A^2^ \| 716.49 \| 716.49 \| 63.10 \| < 0.0001 \| \| B^2^ \| 7.23 \| 7.23 \| 0.64 \| 0.4433 \| \| C^2^ \| 36.66 \| 36.66 \| 3.23 \| 0.1026 \| \| Residual \| 113.54 \| 11.35 \|  \|  \| \| Lack of Fit \| 14.93 \| 2.99 \| 0.15 \| 0.9706 \| \| R^2^ \|  \| 0.98 \|  \|  \| \| adj. R^2^ \|  \| 0.97 \|  \|  \| | **Table S2**: The results of ANOVA analysis for the response of glucose yield of alkaline pretreatment   \|  \| Sum of \| Mean \| F \| p-value \| \| --- \| --- \| --- \| --- \| --- \| \| Source \| Squares \| Square \| Value \|  \| \| Model \| 45603.82 \| 5067.09 \| 111.54 \| < 0.0001 \| \| A-Alkaline \| 29181.47 \| 29181.47 \| 642.38 \| < 0.0001 \| \| B-Time \| 367.55 \| 367.55 \| 8.09 \| 0.0174 \| \| C-Temp \| 102.76 \| 102.76 \| 2.26 \| 0.1635 \| \| AB \| 114.51 \| 114.51 \| 2.52 \| 0.1434 \| \| AC \| 1.13 \| 1.13 \| 0.02 \| 0.8781 \| \| BC \| 80.64 \| 80.64 \| 1.78 \| 0.2123 \| \| A^2^ \| 7869.78 \| 7869.78 \| 173.24 \| < 0.0001 \| \| B^2^ \| 70.48 \| 70.48 \| 1.55 \| 0.2413 \| \| C^2^ \| 128.32 \| 128.32 \| 2.82 \| 0.1237 \| \| Residual \| 454.27 \| 45.43 \|  \|  \| \| Lack of Fit \| 255.36 \| 51.07 \| 1.28 \| 0.3953 \| \| R^2^ \|  \| 0.99 \|  \|  \| \| adj. R^2^ \|  \| 0.98 \|  \|  \| |
| --- | --- | --- | --- | --- | --- | --- | --- | --- | --- | --- | --- | --- | --- | --- | --- | --- | --- | --- | --- | --- | --- | --- | --- | --- | --- | --- | --- | --- | --- | --- | --- | --- | --- | --- | --- | --- | --- | --- | --- | --- | --- | --- | --- | --- | --- | --- | --- | --- | --- | --- | --- | --- | --- | --- | --- | --- | --- | --- | --- | --- | --- | --- | --- | --- | --- | --- | --- | --- | --- | --- | --- | --- | --- | --- | --- | --- | --- | --- | --- | --- | --- | --- | --- | --- | --- | --- | --- | --- | --- | --- | --- | --- | --- | --- | --- | --- | --- | --- | --- | --- | --- | --- | --- | --- | --- | --- | --- | --- | --- | --- | --- | --- | --- | --- | --- | --- | --- | --- | --- | --- | --- | --- | --- | --- | --- | --- | --- | --- | --- | --- | --- | --- | --- | --- | --- | --- | --- | --- | --- | --- | --- | --- | --- | --- | --- | --- | --- | --- | --- | --- | --- | --- | --- | --- | --- | --- | --- | --- | --- | --- | --- |
|  |  |
| **Table S3**: The results of ANOVA analysis for the response of ABTS antioxidant activity of acid pretreatment   \|  \| Sum of \| Mean \| p-value \| \| --- \| --- \| --- \| --- \| \| Source \| Squares \| Square \|  \| \| Model \| 1887.92 \| 145.22 \| <0.0001 \| \| A-Acid \| 2.69 \| 2.69 \| 0.4352 \| \| B-Time \| 76.57 \| 76.57 \| 0.0043 \| \| C-Temp \| 178.85 \| 178.85 \| 0.0005 \| \| AB \| 18.12 \| 18.12 \| 0.0730 \| \| AC \| 49.80 \| 49.80 \| 0.0114 \| \| BC \| 23.53 \| 23.53 \| 0.0482 \| \| A^2^ \| 220.35 \| 220.35 \| 0.0003 \| \| B^2^ \| 7.46 \| 7.46 \| 0.2131 \| \| C^2^ \| 0.26 \| 0.26 \| 0.8028 \| \| ABC \| 7.33 \| 7.33 \| 0.2165 \| \| A^2^B \| 0.01 \| 0.01 \| 0.9626 \| \| AB^2^ \| 23.16 \| 23.16 \| 0.0495 \| \| AC^2^ \| 74.04 \| 74.04 \| 0.0046 \| \| Residual \| 23.07 \| 3.84 \|  \| \| Lack of Fit \| 12.83 \| 12.83 \| 0.0543 \| \| R^2^ \|  \| 0.99 \|  \| \| adj. R^2^ \|  \| 0.96 \|  \| | **Table S4**: The results of ANOVA analysis for the response of ABTS antioxidant activity of alkaline pretreatment   \|  \| Sum of \| Mean \| p-value \| \| --- \| --- \| --- \| --- \| \| Source \| Squares \| Square \|  \| \| Model \| 2326.38 \| 193.86 \| < 0.0001 \| \| A-Alkaline \| 18.44 \| 18.44 \| 0.0116 \| \| B-Time \| 89.19 \| 89.19 \| 0.0001 \| \| C-Temp \| 93.70 \| 93.70 \| 0.0001 \| \| AB \| 3.47 \| 3.47 \| 0.1846 \| \| AC \| 4.46 \| 4.46 \| 0.1394 \| \| BC \| 2.50 \| 2.50 \| 0.2520 \| \| A^2^ \| 128.80 \| 128.80 \| < 0.0001 \| \| B^2^ \| 0.00 \| 0.00 \| 0.9777 \| \| C^2^ \| 15.43 \| 15.43 \| 0.0173 \| \| ABC \| 13.39 \| 13.39 \| 0.0233 \| \| AB^2^ \| 38.92 \| 38.92 \| 0.0017 \| \| AC^2^ \| 23.63 \| 23.63 \| 0.0064 \| \| Residual \| 11.22 \| 1.60 \|  \| \| Lack of Fit \| 5.66 \| 2.83 \| 0.1731 \| \| R^2^ \|  \| 0.99 \|  \| \| adj. R^2^ \|  \| 0.99 \|  \| |
|  |  |
| **Table S5**: The results of ANOVA analysis for the response of FRAP antioxidant activity of acid pretreatment   \|  \| Sum of \| Mean \| p-value \| \| --- \| --- \| --- \| --- \| \| Source \| Squares \| Square \|  \| \| Model \| 3159.14 \| 243.01 \| < 0.0001 \| \| A-Acid \| 19.56 \| 19.56 \| 0.0070 \| \| B-Time \| 467.25 \| 467.25 \| < 0.0001 \| \| C-Temp \| 360.62 \| 360.62 \| < 0.0001 \| \| AB \| 3.11 \| 3.11 \| 0.1604 \| \| AC \| 8.61 \| 8.61 \| 0.0372 \| \| BC \| 7.11 \| 7.11 \| 0.0517 \| \| A^2^ \| 261.24 \| 261.24 \| < 0.0001 \| \| B^2^ \| 175.52 \| 175.52 \| < 0.0001 \| \| C^2^ \| 62.34 \| 62.34 \| 0.0004 \| \| ABC \| 83.98 \| 83.98 \| 0.0002 \| \| A^2^B \| 60.71 \| 60.71 \| 0.0004 \| \| A^2^C \| 18.28 \| 18.28 \| 0.0081 \| \| AB^2^ \| 11.54 \| 11.54 \| 0.0215 \| \| Residual \| 7.27 \| 1.21 \|  \| \| Lack of Fit \| 1.12 \| 1.12 \| 0.3834 \| \| R^2^ \|  \| 0.99 \|  \| \| adj. R^2^ \|  \| 0.99 \|  \| | **Table S6**: The results of ANOVA analysis for the response of FRAP antioxidant activity of alkaline pretreatment   \|  \| Sum of \| Mean \| p-value \| \| --- \| --- \| --- \| --- \| \| Source \| Squares \| Square \|  \| \| Model \| 2550.55 \| 231.87 \| < 0.0001 \| \| A-Alkaline \| 1007.45 \| 1007.45 \| < 0.0001 \| \| B-Time \| 14.36 \| 14.36 \| 0.1958 \| \| C-Temp \| 42.17 \| 42.17 \| 0.0419 \| \| AB \| 129.33 \| 129.33 \| 0.0029 \| \| AC \| 32.44 \| 32.44 \| 0.0667 \| \| BC \| 10.24 \| 10.24 \| 0.2675 \| \| A^2^ \| 18.08 \| 18.08 \| 0.1519 \| \| B^2^ \| 6.83 \| 6.83 \| 0.3588 \| \| C^2^ \| 1.21 \| 1.21 \| 0.6930 \| \| A^2^B \| 79.43 \| 79.43 \| 0.0105 \| \| AC^2^ \| 347.40 \| 347.40 \| 0.0001 \| \| Residual \| 57.66 \| 7.21 \|  \| \| Lack of Fit \| 36.26 \| 12.09 \| 0.1463 \| \| R^2^ \|  \| 0.98 \|  \| \| adj. R^2^ \|  \| 0.95 \|  \| |
|  |  |
| **Table S7**: The results of ANOVA analysis for the response of DPPH antioxidant activity of acid pretreatment   \|  \| Sum of \| Mean \| p-value \| \| --- \| --- \| --- \| --- \| \| Source \| Squares \| Square \|  \| \| Model \| 483.00 \| 40.25 \| < 0.0001 \| \| A-Acid \| 0.23 \| 0.23 \| 0.3514 \| \| B-Time \| 164.13 \| 164.13 \| < 0.0001 \| \| C-Temp \| 59.19 \| 59.19 \| < 0.0001 \| \| AB \| 1.22 \| 1.22 \| 0.0567 \| \| AC \| 0.10 \| 0.10 \| 0.5415 \| \| BC \| 0.20 \| 0.20 \| 0.3817 \| \| A^2^ \| 40.22 \| 40.22 \| < 0.0001 \| \| B^2^ \| 26.68 \| 26.68 \| < 0.0001 \| \| C^2^ \| 6.77 \| 6.77 \| 0.0010 \| \| ABC \| 17.76 \| 17.76 \| < 0.0001 \| \| A^2^C \| 2.90 \| 2.90 \| 0.0098 \| \| AB^2^ \| 13.48 \| 13.48 \| 0.0001 \| \| Residual \| 1.65 \| 0.24 \|  \| \| Lack of Fit \| 1.10 \| 0.55 \| 0.0620 \| \| R^2^ \|  \| 0.99 \|  \| \| adj. R^2^ \|  \| 0.99 \|  \| | **Table S8**: The results of ANOVA analysis for the response of DPPH antioxidant activity of alkaline pretreatment   \|  \| Sum of \| Mean \| p-value \| \| --- \| --- \| --- \| --- \| \| Source \| Squares \| Square \|  \| \| Model \| 1247.86 \| 178.27 \| < 0.0001 \| \| A-Alkaline \| 973.85 \| 973.85 \| < 0.0001 \| \| B-Time \| 24.60 \| 24.60 \| < 0.0001 \| \| C-Temp \| 65.46 \| 65.46 \| < 0.0001 \| \| AB \| 4.40 \| 4.40 \| 0.0182 \| \| AC \| 8.90 \| 8.90 \| 0.0022 \| \| A^2^ \| 69.54 \| 69.54 \| < 0.0001 \| \| C^2^ \| 5.70 \| 5.70 \| 0.0090 \| \| Residual \| 7.07 \| 0.59 \|  \| \| Lack of Fit \| 5.12 \| 0.73 \| 0.2515 \| \| R^2^ \|  \| 0.99 \|  \| \| adj. R^2^ \|  \| 0.99 \|  \| |

All models were significant at the *p* < 0.0001, which indicated that the models were significant with high confidence levels of more than 99.99%. All the lack-of-fit values were insignificant, suggesting that the established model is adequate for the prediction. All the R^2^ and adj. R^2^ values were higher than 0.80, which indicated that there was a close agreement between experimental results and predicted values. All the R^2^ were in good agreement with their adjusted R^2^, imply a good adjustment of the theoretical values to the experimental data from these models.

**Table S1 and S2** showed the results of ANOVA analysis for the response of glucose yields of acid and alkaline pretreatment, respectively. The final empirical quadratic model in terms of code value were shown as follows:

For acid pretreatment；

Glu = 162.10 + 16.18*A* + 6.46*B* + 10.61*C* - 2.53*AB* - 1.09*AC* + 0.59*BC* - 7.99*A*2 - 0.77*B*2 + 1.83*C*2

For alkaline pretreatment:

Glu = 266.49 + 51.07*A* + 5.17*B* + 2.84*C* - 3.33*AB* - 0.37*AC* - 3.17*BC* - 26.48*A*2 - 2.41*B*2 - 3.42*C*2

Where A, B and C are the acid or alkaline concentration, time and temperature, respectively.

The significance of coefficient of fitted model was evaluated by using the *p*-value. For acid pretreatment, the terms (*A*, *B*, *C*, *AB* and *A*^2^) were found to significantly affect glucose yield (*p*<0.05). For alkaline pretreatment, the terms (*A*, *B* and *A*^2^) had significant effects on glucose yield (*p*<0.05).

**Table S3 and S4** showed the results of ANOVA analysis for the response of the ABTS antioxidant activity of the supernatant of acid and alkaline pretreatment, respectively. The final empirical quadratic model in terms of code value were shown as follows:

For acid pretreatment:

ABTS = 48.52 - 1.78*A* + 3.94*B* + 3.74*C* - 1.50*AB* - 2.49*AC* + 1.72*BC* - 8.06*A*2 - 0.88*B*2 + 0.17*C*2 - 0.96*ABC* + 0.055*A*2*B* + 4.86*AB*2 + 6.98*AC*2

For alkaline pretreatment:

ABTS = 53.91 + 4.64*A* + 2.63*B* + 2.71*C* + 0.66*AB* + 0.75*AC* - 0.56*BC* - 6.15*A*2 - 0.012*B*2 + 1.33*C*2 - 1.29*ABC* + 5.33*AB*2 + 3.24*AC*2

Where A, B and C are the acid or alkaline concentration, time and temperature, respectively.

The significance of coefficient of fitted model was evaluated by using the *p*-value. For acid pretreatment, the terms (*B*, *C*, *AB*, *AC*, *BC*, *A*^2^, *AB*^2^ and *AC*^2^) were found to significantly affect the ABTS antioxidant activity of the supernatant (*p*<0.05). For alkaline pretreatment, the terms (*A*, *B*, *C*, *A*^2^, *C*^2^, *ABC*, *AB*^2^ and *AC*^2^) had significant effects on the ABTS antioxidant activity of the supernatant (*p*<0.05).

**Table S5 and S6** showed the results of ANOVA analysis for the response of the FARP antioxidant activity of the supernatant of acid and alkaline pretreatment, respectively. The final empirical quadratic model in terms of code value were shown as follows:

For acid pretreatment:

FRAP = 41.15 + 4.41*A* + 9.14*B* + 8.70*C* - 0.60*AB* - 1.04*AC* + 0.94*BC* - 8.66*A*2 - 4.15*B*2 - 2.67*C*2 - 3.24*ABC* - 3.66*A*2B - 2.47*A*2C + 3.34*AB*2

For alkaline pretreatment:

FRAP = 52.41 + 22.41*A* + 1.59*B* + 1.82*C* + 3.91*AB* - 2.01*AC* + 1.13*BC* - 1.59*A*2 - 0.80 *B*2 - 0.34*C*2 + 4.32*A*2*B* - 14.72*AC*2

The significance of coefficient of fitted model was evaluated by using the *p*-value. For acid pretreatment, the terms (*A*, *B*, C, *AC*, *A*^2^, *B*^2^, *C*^2^, *ABC*, *A*^2^*B*, *A*^2^*C* and *AB*^2^) were found to significantly affect the FARP antioxidant activity of the supernatant (*p*<0.05). For alkaline pretreatment, the terms (*A*, *C*, *AB*, *A*^2^*B* and *AC*^2^) had significant effects on the FARP antioxidant activity of the supernatant (*p*<0.05).

**Table S7 and S8** showed the results of ANOVA analysis for the response of the DPPH antioxidant activity of the supernatant of acid and alkaline pretreatment, respectively. The final empirical quadratic model in terms of code value were shown as follows:

For acid pretreatment

DPPH= 19.14 - 0.45*A* + 3.48*B* + 3.53*C* - 0.37*AB* - 0.11*AC* - 0.16*BC* - 3.33*A*2 - 1.52*B*2 - 0.87*C*2 - 1.49*ABC* -0.99*A*2C + 3.12*AB*2

For alkaline pretreatment:

DPPH = 33.30 + 9.04*A* + 1.33*B* + 2.26*C* + 0.65*AB* + 1.06*AC* - 2.42*A*2 + 0.71*C*2

The significance of coefficient of fitted model was evaluated by using the *p*-value. For acid pretreatment, the terms (*B*, *C*, *A*^2^, *B*^2^, *C*^2^, *ABC*, *A*^2^*C* and *AB*^2^) were found to significantly affect the DPPH antioxidant activity of the supernatant (*p*<0.05). For alkaline pretreatment, the terms (*A*, *B*, *C*, *AB*, *AC*, *A*^2^, *C*^2^) had significant effects on the DPPH antioxidant activity of the supernatant (*p*<0.05).

**Table S9. Experimental data of the validation of predicted values at optimal pretreatment conditions**

| Dependent | Predicted | Experimental | % Difference |
| --- | --- | --- | --- |
| variables | value | value | (CV) |
| Acid pretreatment | |  |  |
| Glu | 185.0 | 188.8 ± 3.7 | 1.45 |
| TP | 11.09 | 11.84 ± 0.11 | 4.61 |
| *p*-CA | 0.47 | 0.44 ± 0.00 | 5.40 |
| FA | 0.84 | 0.74 ± 0.00 | 8.91 |
| ABTS | 56.13 | 55.24 ± 0.63 | 1.19 |
| FRAP | 48.25 | 43.85 ± 0.55 | 6.76 |
| DPPH | 22.56 | 20.13 ± 0.51 | 7.97 |
| Acid pretreatment | |  |  |
| Glu | 285.9 | 285.7 ± 5.4 | 0.05 |
| TP | 27.33 | 26.79 ± 0.47 | 1.39 |
| *p*-CA | 7.24 | 6.98 ± 0.03 | 2.64 |
| FA | 3.87 | 3.84 ± 0.01 | 0.56 |
| ABTS | 68.51 | 67.92 ± 0.21 | 0.62 |
| FRAP | 67.22 | 67.33 ± 2.92 | 0.11 |
| DPPH | 45.63 | 45.51 ± 0.75 | 0.16 |

Glu (glucose yield: mg g^-1^), TP (total polyphenol yield: mg GAE g^-1^), *p*-CA (*p*-coumaric acid yield: mg g^-1^), and FA (ferulic acid yield: mg g^-1^). ABTS, FRAP, and DPPH (antioxidant activity: μmol AAE g^-1^)
